# Repetitive transcranial magnetic stimulation (rTMS) of the primary motor cortex for treating facial neuropathic pain – preliminary results of a randomized, sham-controlled, cross-over study

**DOI:** 10.1186/1744-8069-10-S1-P6

**Published:** 2014-12-15

**Authors:** Magdalena Lang, Roi Treister, Max Klein, Anne Louise Oaklander

**Affiliations:** 1 Department of Neurology, Massachusetts General Hospital, Harvard Medical School, Boston, MA 02114, USA; 2 Departments of Pathology (Neuropathology), Massachusetts General Hospital, Boston, MA 02114, USA

## Background

Repetitive transcranial magnetic stimulation (rTMS) noninvasively triggers action potentials in underlying brain cortex intended for therapeutic benefit. Currently marketed for refractory depression, rTMS is under investigation for treating several neurological conditions including pain. Facial neuropathic pain (NP) appears to respond well to rTMS [1]. The primary motor cortex (M1) is established as the best site to stimulate to relieve pain, but the best target within M1 remains unknown [1,2]. MRI guidance of rTMS permits repeated precise application to specific targets. The aim of this study was to compare pain relief obtained by applying MRI-guided rTMS to the M1 representation of subjects’ painful face vs. to adjacent M1 cortex innervating their non-painful hand.

## Materials and methods

In this ongoing randomized, single-blinded, placebo-controlled, cross-over study, eight adults with facial NP provided informed consent for head MRI and rTMS. Subjects undertook the 3 study phases in random order. In one phase, rTMS was applied to the contralateral M1 representation of their painful face. In the others, rTMS was applied to the adjacent hand area, or sham-rTMS was administered to the hand area using a spacer below the coil. In each phase, 1500 stimuli were administered using a Nexstim NBS 3.2 with a double 70mm coil. Five-second trains of 10 Hz pulses were applied at 80% of resting motor threshold with 55 second inter-train intervals. Each study-phase involved recording baseline data for 3 days, 5 consecutive daily sessions of rTMS, followed by 3 washout weeks. The primary outcome was change in the 0-10 numeric pain rating score. Secondary outcomes measured physical and mental functioning using the SF-36, McGill pain questionnaire and Beck depression Inventory.

## Results

Among the 8 subjects studied so far, 4 had V1 postherpetic neuralgia, 2 had ocular NP, and one each had trigeminal neuralgia and idiopathic facial pain. Reductions in mean pain intensity were >20% on treatment days 3-5 during both active treatments, but not during sham treatment. Pain reverted to baseline within two weeks after ending treatment. 3/8 subjects had pain reductions >30% when the cortex innervating their hand was stimulated and 2/8 had reductions >30% when cortex innervating their face was stimulated. Secondary outcomes did not change and there were no significant adverse events.

## Conclusions

These preliminary results corroborate the efficacy of rTMS for facial NP. Recruitment is ongoing to test the hypothesis that stimulating adjacent healthy cortex may relieve pain better than stimulating cortex representing the painful face [3].

## Disclosures

None of the authors have any conflicts of interest to disclose.
